# Determining factors affecting the user's intention to disclose privacy in online health communities: a dual-calculus model

**DOI:** 10.3389/fpubh.2023.1109093

**Published:** 2023-07-19

**Authors:** Zhuo Sun, Guoquan Zang, Zongshui Wang, Shuang Ge, Wei Liu, Kaiyang Wang

**Affiliations:** ^1^School of Information Management, Zhengzhou University, Zhengzhou, China; ^2^School of Politics and Public Administration, Zhengzhou University, Zhengzhou, China; ^3^School of Economics and Management, Beijing Information S&T University, Beijing, China; ^4^Business School, China University of Political Science and Law, Beijing, China; ^5^School of Economics and Management, China University of Petroleum (Huadong), Qingdao, China; ^6^Business School, Zhengzhou University, Zhengzhou, China

**Keywords:** risk calculus, privacy calculus, privacy disclosure, dual-calculus model, online health communities

## Abstract

**Background:**

As a new type of medical service application for doctor-patient interaction, online health communities (OHCs) have alleviated the imbalance between the supply and demand of medical resources in different regions and the problems of “difficult and expensive access to medical care”, but also raised the concern of patients about the risk of disclosure of their health privacy information.

**Methods:**

In this study, a dual-calculus model was developed to explore users' motivation and decision-making mechanism in disclosing privacy information in OHCs by combining risk calculus and privacy calculus theories.

**Results:**

In OHCs, users' trust in physicians and applications is a prerequisite for their willingness to disclose health information. Meanwhile, during the privacy calculation, users' perceived benefits in OHCs had a positive effect on both trust in doctors and trust in applications, while perceived risks had a negative effect on both trusts in doctors and trust in applications. Furthermore, in the risk calculation, the perceived threat assessment in OHCs had a significant positive effect on perceived risk, while the response assessment had a significant negative effect on perceived risk, and the effect of users' trust in physicians far exceeded the effect of trust in applications. Finally, users' trust in physicians/applications is a mediating effect between perceived benefits/risks and privacy disclosure intentions.

**Conclusion:**

We combine risk calculus and privacy calculus theories to construct a dual-calculus model, which divides trust into trust in physicians and trust in applications, in order to explore the intrinsic motivation and decision-making mechanism of users' participation in privacy disclosure in OHCs. On the one hand, this theoretically compensates for the fact that privacy computing often underestimates perceived risk, complements the research on trust in OHCs, and reveals the influencing factors and decision transmission mechanisms of user privacy disclosure in OHCs. On the other hand, it also provides guidance for developing reasonable privacy policies and health information protection mechanisms for platform developers of OHCs.

## Introduction

With the continued spread of COVID-19 worldwide and the increasing number of confirmed cases, public health has become a major issue that must be faced and coordinated by all countries. However, the contradiction between rapid growth of healthcare demand and the shortage and unbalanced development of healthcare resources has become evident throughout the world, and China is no exception. This makes the rapid development of health care and the effective allocation of health care resources an urgent issue that the health care system must address. According to the China Health Statistical Yearbook 2021, the problem of uneven development between urban and rural areas in the China healthcare industry is more prominent, and the level of allocation of medical infrastructure varies greatly. By the end of 2021, the number of practicing doctors and practicing assistant doctors per 1,000 population in China was 3.04. Among them, the ratio is 4.17 in urban areas but only 1.03 in rural areas. (1) Under the influence of “Internet + Healthcare”, OHCs have become one of the effective ways to alleviate the above problems. With the interpenetration of online social networks and health care, and the increasingly urgent demand for personalized, diversified and real-time medical services, online medical communities, represented by “Ping-an Good Doctor, Good Doctor Online, Chunyu Doctor, DXY and Alibaba Health”, have grown significantly (2). Through OHCs, patients can get medical information, communicate with physicians, and get treatment advice online anytime and anywhere, which not only breaks the limitation of time and space but also reduces the cost of medical treatment for users. To some extent, OHCs have alleviated the imbalance between the supply and demand for medical resources in different regions and the problems that have long plagued our residents, such as the difficulty and high cost of seeing a doctor, and have become an important way for Internet users to understand their health status and obtain health information (3). Compared with traditional hospitals that provide only a small amount of information on hospital rank, physicians' titles and areas of expertise, online physician-patient consultation platforms can provide detailed physician information as well as patient evaluation information. This information can alleviate the information asymmetry between physicians-patients to a certain extent, reduce the uncertainty of medical services, and thus provide guidance for patients to make reasonable consultation decisions (4).

In OHCs, patients are often required to provide detailed personal information (e.g., name, gender, age, and contact information) and health information (symptoms of illness, personal history of illness, family history of illness, and medical reports) to participate in OHCs. On one hand, this can provide patients with a good personalized service experience and rich medical resources (such as one-on-one communication with doctors, real-time consultation, health management and online medical report interpretation), and provide experience for other patients suffering from similar diseases. On the other hand, it also helps to provide doctors with more first-hand clinical case data, thus laying a solid foundation for improving diagnosis and treatment (5). However, the personal information provided by patients participating in OHCs is uploaded to the cloud for analysis, which increases the risk of user privacy leakage. The leakage of personal health records such as allergy medications, family medical history and imaging reports can cause serious privacy violations and personal safety problems for patients (6). There have been numerous incidents of user health information leaks in recent years, with reports that Over 40 million people in the United States had their personal health information exposed in data breaches (7), which has also raised their concerns about health information breaches. So, what factors influence users' privacy disclosure intentions in OHCs? What are the decision transmission mechanisms of user privacy disclosure? These questions deserve to trigger extensive thinking in both academic and practical circles.

However, a review of prior research on user privacy disclosure behaviors in OHCs revealed the following three research gaps remain. First, although past studies have discussed various aspects of user privacy disclosure behaviors in different contexts, less attention has been paid to patient health information disclosure intentions/behaviors in the context of OHCs. With the explosive growth of Internet users and the transformation of users' health management style in the post-epidemic era, OHCs have become increasingly popular among the majority of Internet users and have become one of the important ways for people to obtain health information. Unlike such scenarios as e-commerce (8), social media (9, 10), online advertising (11), social networks (12), and mobile apps (13), the private health information disclosed by users in OHCs is relatively unique and very important, as evidenced by the serious consequences of user health information violations (e.g., discrimination and social stigma). For example, when people diagnosed with infectious diseases such as HIV, viral hepatitis, and tuberculosis seek medical help from doctors in OHCs, they can be discriminated against by employers and even lose their jobs if their personal information is compromised. Also, lesbian, gay, bisexual, and transgender people may encounter discrimination when the sexual orientation they choose on OHC is exposed to their friends or family. Privacy disorders may be discriminated against and treated unfairly (2). Furthermore, unlike other online communities, users receive more social support than monetary rewards, discounts, or social benefits in OHCs (14). Thus, although users' disclosure of private information can lead to rich material benefits, social benefits, and service experiences in other online communities, these benefits are not the main drivers for users to disclose health information in OHCs. Therefore, further deeper exploration of the intrinsic motivations and potential risks of users' self-disclosure of health information in the OHCs context is needed.

Second, although there have been many studies on the relationship between perceived privacy risks and disclosure intentions, few have explored the antecedents of perceived privacy risks and combined the three to analyze patient decisions to disclose health information in OHCs. Phelps et al. (15) argued that the causes of privacy concerns/risk must be identified to investigate privacy issues. By identifying the antecedents of perceived privacy risk, patients in OHCs can be helped to perceived benefits and perceived risks to be measured effectively, thus reducing anxiety and uncertainty of users in disclosing health information (15). Most studies have been conducted to analyze users' motivation for privacy disclosure using privacy calculus, which argues that privacy disclosure decisions are made based on the difference between perceived expected benefits and risks. However, Li (16) argued that under the guidance of privacy calculus, users tend to pay more attention to perceived benefits and near-term benefits while often ignoring potential risks and long-term losses when making privacy disclosures (16). Moreover, it is difficult to obtain information related to the assessment of privacy risks when making privacy decisions due to their own perceptions or information asymmetry. These may lead to users' inability to perform a complete risk-benefit calculation and their apparent perception of benefits and ignorance of privacy risks, thus tending to use perceived benefits as the main consideration for privacy decisions (17). However, according to PMT, users' perception of risk is determined by a combination of their assessment of external threats (threat assessment) and their own ability (response assessment), which makes the use of privacy computation theory alone not an accurate reflection of users' perception of risk when making privacy decisions. The balance between the threat assessment and the response assessment is called the risk calculation. Therefore, introducing risk calculations into the original model of privacy calculations and patient disclosure intentions is essential to gain a comprehensive understanding of patients' motivations for disclosing health information in OHCs.

Third, the existing research has mainly focused on the effects of trust in the applications/services, as well as its predictors (2), while few studies have multidimensionally conceptualization. For example, trust between patients and doctors, between patients and technology, or between patients and applications. Unlike physical goods transactions or online commerce transactions, healthcare services are an intangible experience, which requires an adequate trust relationship between the user and the platform in order to reduce the user's risk perception and uncertainty (18, 19). In this process, the most important role of OHCs applications is to reduce users' perception of near-term risks and assessment of distant threats through strong corporate endorsement and good social reputation, thus enhancing users' initial trust in applications (20, 21). In addition, the specialization of healthcare services has increased the information asymmetry between physicians and patients, which makes patients more dependent on initial trust building (22). Trust in physicians and applications can reduce certain risk perceptions and thus make patients willing to disclose personal information. Therefore, it is necessary to explore the transmission mechanisms of patients' health information disclosure behavior in OHCs by dividing trust into trust in physicians and trust in applications.

In summary, to bridge the above three research gaps, this paper combines risk calculation theory and privacy calculation theory to construct a dual privacy calculation model, dividing trust into trust in doctors and trust in applications to explore the intrinsic motivation and decision-making mechanism of users' participation in privacy disclosure in OHCs. On the one hand, this theoretically compensates for the fact that privacy computing often underestimates perceived risk, complements the research on trust in OHCs, and reveals the influencing factors and decision transmission mechanisms of user privacy disclosure in OHCs. On the other hand, it also provides guidance for developing reasonable privacy policies and health information protection mechanisms for platform developers of OHCs.

## Literature review and hypothesis development

### Privacy disclosures and OHCs

We review the previous decision-making process on user privacy disclosure behavior and classify it into three categories in the context of OHCs, as follows:

First, “risk-benefit” calculations based on rational hypotheses. In the process of information exchange, users' privacy disclosure intentions or actual behaviors are positively influenced by expected benefits (social relationships, personalized services, discounts, financial rewards, and convenience, etc.) and negatively influenced by perceived risks (e.g., privacy leakage, privacy invasion, inappropriate access, and secondary use, etc.). Users make the decision to disclose privacy information when the perceived benefits of privacy disclosure outweigh the perceived risks. Conversely, no disclosure is made (23). For example, Zhang et al. (14) explored the antecedents and consequences of privacy concerns in OHCs by integrating privacy computation theory and protection motivation theory. They used informational and emotional support as a proxy variable for perceived benefits and health information privacy concerns as a proxy variable for perceived risks. The results showed that perceived benefits had a significant negative effect on privacy concerns, while perceived risks exerted the opposite effect (14).

Second, the biased estimation of privacy decision risk due to limited rationality. Users are bound by multiple cognitive biases in decision making and are unable to make fully rational privacy decisions. Because limited rationality limits their ability to process the information they obtain, they rely on simplified mental models, approximation strategies, and heuristics when making decisions. Moreover, even if users have access to complete information and develop optimal privacy decision strategies, they may still deviate from rational strategies (24). For example, based on heuristic-systematic model (HSM), information ecology theory, privacy trade-off and self-efficacy theory, Gao et al. constructed a model. They found that information quality and emotional support had indirect effects on heuristic and systematic effects on information processing by users of OHCs. Indirect effects on heuristic and systematic information processing, and these effects were mediated by privacy concerns and self-efficacy (25).

Third, the perceived benefits are dominant and lack the assessment of privacy decision risks. In this case, users have almost no access to all the information relevant to assessing privacy risks due to their own perceptions or information asymmetry, etc. This leads to the inability to perform a complete risk-benefit calculation and ignore privacy risks, thus tending to use perceived benefits as the main consideration for decision making (17). This is consistent with the findings of Barth and De Jong (17). For example, building on the dual-process model (i.e., conscious process and unconscious process), the authors discussed the influences of social support received in two ways: social support received by initiating threads (direct social support receipt) and social support received by being exposed to the threads The authors discussed the influences of social support received in two ways: social support received by initiating threads (direct social support receipt) and social support received by being exposed to the threads (indirect social support receipt).

In general, existing research mostly underestimates the negative impact and potential threat of perceived risk to patients in OHCs from a privacy calculus perspective, which is less applicable to scenarios like OHCs where health information is highly valued. Also, this is not conducive to a comprehensive understanding of the motivational role pathways of users in OHCs for health privacy information disclosure. Therefore, further research is needed on the issue of how users make privacy disclosure decisions in OHCs.

### Perceived risk from a risk calculation perspective

The process of risk calculus stems from the balance between threat appraisal and coping appraisal in protection motive theory, which measures the net risk (such as net privacy risks) perceived by users when processing online transactions (16). Protective Motivation Theory (PMT) was used to explore whether protective motivation formed by people's fearful assessment of risk information influences the protective behavior of the self. The protection motivation theory proposes two cognitive processes that individuals perform to cope with threats: threat appraisal and coping appraisal. In general, threat appraisal is a process that assesses the severity and susceptibility of threats, including the perceived importance of privacy information (26), perceived severity of privacy breach (27), and perceived vulnerability of privacy response (28), whereas coping appraisal is a process that assesses the effectiveness of protective responses and the self-efficacy of individuals exposed to threats (29), including self-efficacy of privacy information (30), and response efficacy (31), perceived control (31), and personality traits (12, 32), etc.

PMT assumes that when an individual is exposed to a threat the possible risk (threat appraisal) and the effectiveness of the protective behavior that can be taken (coping appraisal) are assessed. That is, the perceived risk of an individual taking adaptive decisions is determined by calculating the difference between the expected risk and the effect of coping, a process known as risk calculus (33). In this study, the risk calculus process of threat appraisal and coping appraisal determines whether individuals will adopt protective behaviors in response to threats. Among them, threat assessment refers mainly to when users perceive a possible risk of physical, psychological, privacy, functional, social, and financial violations in the online health community. Since users question the reliability and trustworthiness of the services and doctors' expertise offered by OHCs due to their poor understanding of them and lack of knowledge about privacy information protection, they usually perceive the effectiveness of the current protective behaviors available to them as low and lack confidence in their ability to take protective countermeasures. At this point, the net risk is higher. Conversely, users generate lower levels of net risk when they know more about and perceive diagnostic services in OHCs applications and physicians as reliable, and when they have more experience with privacy protection. At this point, users are more willing to engage in high-risk transactions when necessary. Therefore, we posit the following two hypotheses:

**Hypothesis 1(H1):** In OHCs, users' threat appraisal has a positive effect on their perceived risks.
**Hypothesis 2(H2):** In OHCs, users' coping appraisal has a negative effect on their perceived risks.

### User trust in physicians/applications from the perspective of privacy calculus

Privacy calculus theory, also known as “behavioral calculus theory,” assumes that individuals or organizations are considering the expected consequences of their actions, which means that individuals weigh the expected benefits and risks when making decisions (34). Previous research has shown that users' disclosure behavior is subject to a combination of perceived benefits and perceived risks. When users make privacy decisions, they tend to share or pass on personal information to third parties if the perceived benefits of disclosing privacy information outweigh the perceived risks; conversely, they do not (35). Hanna et al. (36) found that although social networks provide users with various privacy settings when they go online, most users believe that the initial reason for choosing such platforms is to establish a more convenient and efficient social relationship with patients. Thus, although users disclose much personal information during use, the perceived benefits in terms of improved physician-patient relationships, reduced wait times, more efficient care, and reduced uncertainty can largely offset concerns about potential risks.

With the continuous improvement of privacy protection laws and platform governance norms, OHCs applications are also moving toward scale, standardization, and standardization, which invariably raises the level of user assessment of the reliability and trustworthiness of their applications. At the same time, doctors in online health community applications are registered with real information and provide detailed profiles, which further reduces users' wariness of doctors (2). In this context, when users can easily use the various convenient services provided by the application on the one hand. On the other hand, they can get to know the personal information of the doctors related to their consultation needs and can have one-on-one communication with them, which can largely improve the users' perception of the personalized services provided by the doctors and their expectation of the results of the consulted problems. The higher the level of personalized service perceived by the user and the degree of expectation of the outcome, the greater his confidence in the application and the doctor to provide satisfactory service and treatment of the disease (6). And when users perceive a risk of possible physical, psychological, privacy, functional, social, and financial violations in an application of an online health community based on their previous experience, they usually choose to stop trusting the website and the doctors on the website to avoid potential losses for the sake of protecting their interests (3). Previous studies have shown that the leakage of users' personal information may become an accomplice to cyber-attacks or even human flesh searches, which can reduce users' trust in social networking platforms. Compared to online transactions in general business contexts, the perceived risks to users in OHCs regarding the health information they disclose will be further magnified. This is because user health information is more directly related to patient safety, life and property, and their own interests. Therefore, although user-provided information can improve the accuracy and quality of online healthcare services, the original level of trust will be reduced when they question the reliability or professionalism of online health community applications or physicians (37). Therefore, the following hypothesis is proposed.

**Hypothesis 3(H3)**: *In OHCs, users' perceived benefits have a positive effect on their trust in physicians*.
**Hypothesis 4(H4)**: *In OHCs, users' perceived benefits have a positive effect on their trust in applications*.
**Hypothesis 5(H5)**: *In OHCs, users' perceived risks have a negative effect on their trust in physicians*.
**Hypothesis 6(H6)**: *In OHCs, users' perceived risks have a negative effect on their trust in applications*.

### Trust theory

Trust theory has been widely studied in sociology, psychology, management, and information resource management, and is considered a key factor influencing the behavior of interpersonal interactions. Trust can be seen as a social resource that constitutes a benchmark for interpersonal communication (38). Trust in transactional relationships has a significant impact on reducing transaction costs, since trust creates positive expectations and reduces the perceived risk of users (38). Trust has been shown not only to enhance users' willingness to share information and the probability of making purchase decisions (39), but also to reduce uncertainty and psychological risk by increasing the psychological distance between patients and physicians (40).

In general, trust indicates people's willingness to rely on or to be vulnerable to other parties (29, 30), and it is often seen as an important factor in building and strengthening relationships and facilitating information exchange among users (41, 42). Previous studies have shown that patients' trust in OHCs and in the physician's hospital rating is projected to the physician through a transmission mechanism. Most medical communities have established a reputation system. That is, an online rating system and online text reviews to reflect the quality of the doctor's online services and treatment results. In particular, the online rating establishes a quantitative measure of other patients' satisfaction with the physician's online treatment. The online rating provides a perceptual assessment of patient satisfaction with the online visit. Together, these two establish the foundation for new patients to gain an understanding of the physician and establish an initial trusting relationship prior to their visit (43). In trust transfer theory, online reviews generated by similar patients will become an important basis for other patients to choose a physician (43). Unlike physical commodity transactions, healthcare services are intangible and must be perceived by patients. The most important role of online healthcare platforms is to reduce the perception of risk and uncertainty so that patients can assess the quality of services and thus build trust (44, 45). Reputation has a positive impact on users' choices. In e-commerce, many scholars have found that a firm reputation can influence users' initial trust and purchase behavior (18, 19). Previous studies have also shown that trust in physicians and applications can reduce users' perceptions of risk, making patients more willing to disclose personal information (2).

However, the existing research has mainly focused on the effects of trust in the applications/services, as well as its predictors (2), and few studies have mostly conceptualized it. For example, trust between patients and doctors, trust between patients and technology, or trust between patients and applications. Based on this, this paper refines the transmission process of different trusts of users in OHCs by dividing them into trust in physicians and trust in applications. We hypothesize the following:

**Hypothesis 7(H7)**: *In OHCs, trust in physicians has a positive effect on their privacy disclosure intentions*.
**Hypothesis 8(H8)**: *In OHCs, users' trust in applications has a positive effect on their privacy disclosure intentions*.
**Hypothesis 9(H9a&H9b&H9c&H9d)**: *In OHCs, users' trust in physicians/applications plays a mediating effect between perceived benefits (H9a&H9b)/perceived risks (H9c&H9d) and privacy disclosure intentions*.

**Figure 1:**
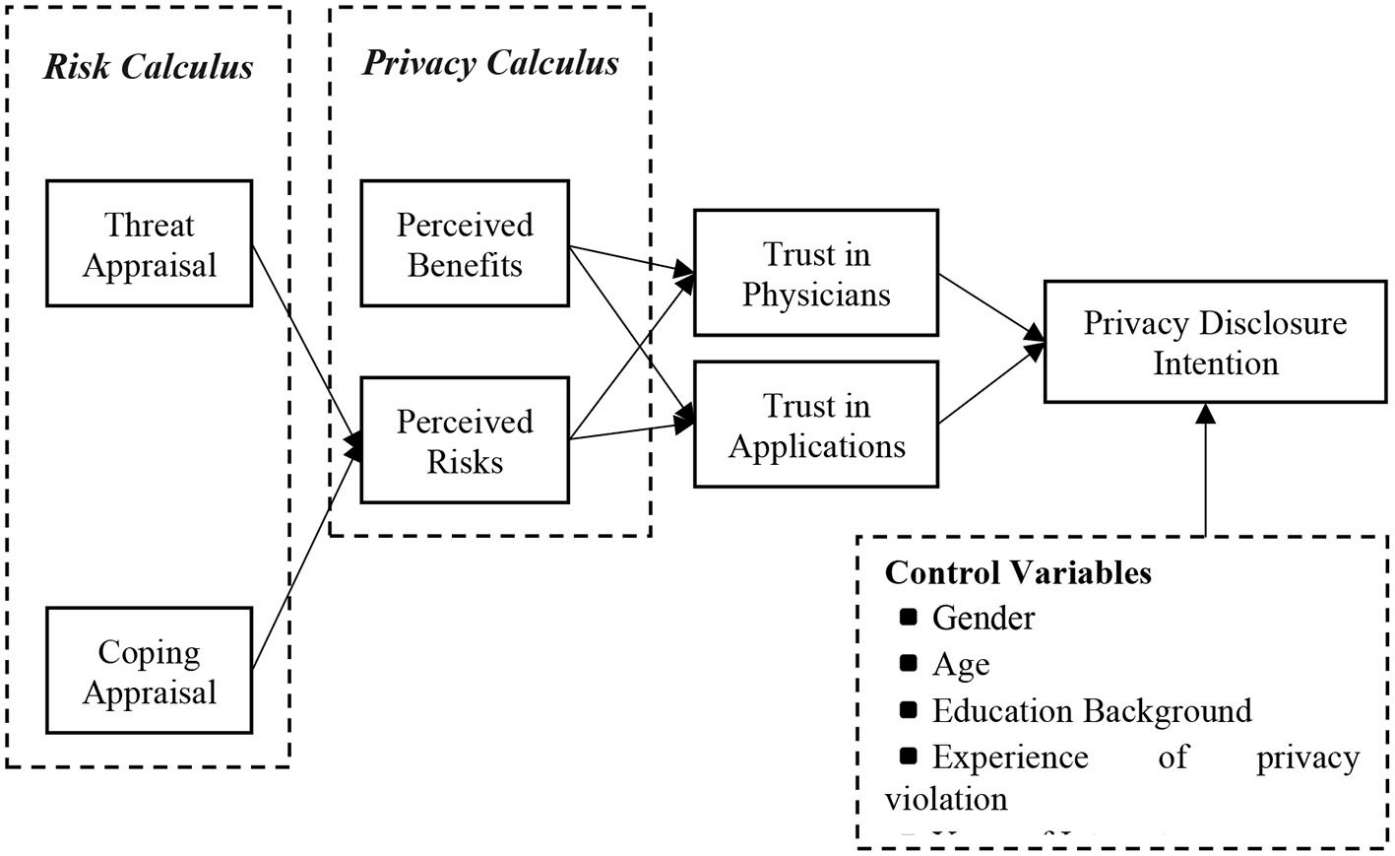
Conceptual model.

Based on the discussion above, we present our conceptual model in Figure 1.

## Research methodology and data collection

### Instrument development

In this study, we used a questionnaire to collect data on Questionnaire Star (https://www.wjx.cn/) to verify whether the hypotheses in the conceptual model were supported. Therefore, we divided the questionnaire into four parts: part 1 is a brief introduction of the questionnaire and notes; part 2 is the basic information about respondents' use of OHCs; part 3 is the measurement questions of each construct in the conceptual model, and part 4 is demographic information. In order to improve the validity and reliability of the scale, a more mature scale was used, and five experts were invited to translate and back-translate the questions several times to ensure the accuracy of the language expression. Furthermore, based on a review of existing studies, sex, age, education, experience with privacy violations and years of Internet use were used as control variables that could have an impact on privacy disclosure intentions. Except for basic information about user participation in OHCs and control variables, all other constructs were measured using a Likert seven-level scale design. “1” represents “strongly disagree” and “7” represents strongly agree. The set and source of the constructs to be measured in the model are shown in Table 1.

**Table 1:** Variable measurement and source.

**Potential variables**	**Questions**	**Source**
Threat appraisal(TA)	If my information were released to unauthorized people, it would be severe	(46)
	If my information were released to unauthorized people, it would be serious	
	If my information were released to unauthorized people, it would be significant	
	My information privacy is at risk of being invaded	
Coping appraisal(CA)	I am able to minimize the risk of disclosing privileged information	(46)
	It is easy for me to take some privacy measures to reduce the risk of privacy violations	
	It is convenient for me to keep my privacy safe through privacy practices	
	It's easy for me to use privacy safeguards to protect my privacy	
Perceived benefits(PB)	I think providing more information will get me more personalized services tailored to my activity context	(47, 48)
	I think providing more information will get more relevant information tailored to my preferences or personal interests.	
	I think providing more information will provide me with the kind of information or service that I might like	
Perceived risks(PR)	Provide individual health information would involve many unexpected problems	(23, 47, 48)
	It would be risky to disclose my health information	
	There would be high potential for loss in disclosing my health information	
Trust in physicians(TP)	The physicians provide reliable information.	(2, 49, 50)
	The physicians in this OHC service keeps promises and commitments.	
	The physicians' behaviors in this OHC service meets my expectations	
Trust in applications(TA)	This mobile medical treatment platform service provider provides reliable information.	(2, 49, 50)
	This mobile medical treatment platform service provider keeps promises and commitments.	
	This mobile medical treatment platform service provider's behavior meets my expectations	
Privacy disclosure intention(PDI)	I am likely to provide my personal health information on OHCs.	(48)
	I am willing to provide my personal health information on OHCs to access relevant services.	
	It is possible for me to provide personal health information on OHCs	

###  Data collection and demographic information

The respondents of this study are those who have used Ping-An Good Doctor (https://www.jk.cn/), Good Doctor Online (https://www.haodf.com/), and Chunyu Doctor (https://www.chunyuyisheng.com/), DXY (https://dxy.com/) and Alibaba Health (https://www.alihealth.cn/) in the last 6 months. This is because the above-mentioned websites are OHCs with larger scale, higher penetration rate, more active people, and higher profitability in China, which are more representative. Meanwhile, to ensure the validity of the questionnaire, 10 researchers engaged in research in the online health community and 25 users who are familiar with the online health community platforms were invited to pre-test the questionnaire before the formal collection. Based on the expert opinions and pretest results, the questionnaire design and language were modified to ensure that all questions were closely related to the measured constructs and clearly differentiated from other constructs to ensure the content validity and discriminant validity of the questionnaire. Data were formally collected using a paid method to increase the motivation of respondents, and the questionnaire was established with response time, content testing, and reverse questions. The survey lasted for 2 months, 425 questionnaires were distributed and 400 valid questionnaires were collected. The sample statistics are shown in Table 2.

**Table 2:** Sample demographics.

**Measure**	**Item**	**Frequency**	**Percentage**	**Measure**	**Item**	**Frequency**	**Percentage**
Platform type	Ping-An Good Doctor	321	80.25%	Age	< 2000	42	10.50%
	Good Doctor	301	75.25%		2000~4000	55	13.75%
	Chunyu Doctor	282	70.50%		4000~6000	98	24.50%
	DXY	253	63.25%		6000~8000	102	25.50%
	Alibaba Health	198	49.50%		>8000	103	25.75%
	Others	34	8.50%	Experience of privacy violation	≤ 2	18	4.50%
Gender	Male	211	52.75%		3~4	101	25.25%
	Female	189	47.25%		5~6	112	28.00%
Educational background	Under a bachelor's degree	37	9.25%		≥7	169	42.25%
	Bachelor's degree	324	81.00%	Years of internet use	≤ 2	36	9.00%
	Master degree or above	39	9.75%		3~4	101	25.25%
					5~6	133	33.25%
					≥7	130	32.50%

## Results

### Common method bias

Common method bias is an artificial covariation between predictor and validated variables caused by the same data source or rater, the same measurement environment, the item context, and the characteristics of the items themselves, thus creating serious confounding of findings and potentially misleading conclusions. Common method bias produces systematic measurement error that can expand or narrow the relationship between constructs, resulting in Type I and Type II bias (51) which can call into question the empirical results of the questionnaire. To avoid the estimation bias caused by spurious correlations, social desirability, and leniency effects, this paper uses the following two methods to test the sample data for the problem of common method bias. First, Harman's one-way test can be used to test for common method bias. An exploratory factor analysis of all latent variables reveals that among the 23 indicators, 5 factors have eigenvalues >1, accounting for 62.21% of the total variance. Meanwhile, the first factor before rotation accounts for only 39.1%, which is < 50% (41, 52). Second, the variance inflation factors (VIF) are all < 3.3, which indicates that there is no serious method of co-linear relationship problem between variables (53) (Table 3). In summary, there is no significant common method bias problem.

**Table 3:** Variance inflation factor (VIF) for internal models.

**construct**	**Trust in physicians**	**Trust in applications**	**Perceived risks**	**Privacy disclosure intention**
Threat appraisal			1.217	
Coping appraisal			1.217	
Perceived benefits	1.600	1.600		
Perceived risks	1.600	1.600		
Trust in physicians				1.083
Trust in applications				1.101
Privacy disclosure intention				

### Assessment of the measurement model reliability and validity

The validity of measurement models is usually divided into two aspects: reliability and validity. Reliability refers to the consistency, stability, and reliability of test results, and is usually expressed in terms of internal consistency. Validity refers to the degree to which a measurement instrument or tool can accurately measure what is being measured. Among them, the reliability test uses Cronbach's alpha and combined reliability with thresholds of 0.7 and 0.5, respectively. The data in Table 4 show that the combined reliability (CR) coefficient and Cronbach's α of all latent variables are >0.7, which indicates that the measurement model has high reliability and good internal consistency (61). Meanwhile, the standardized factor loadings of each measurement term were > 0.7, and the average variance extracted (AVE) of each variable was higher than 0.5, which indicated that the measurement model had good convergent validity (54). In addition, the square root of the AVE for each variable was greater than the correlation coefficient between the latent variables (Table 5) (42), which indicates good discriminant validity between the latent variables.

**Table 4:** Reliability and validity analysis results.

**Construct**	**Item**	**Standardized loadings**	**AVE**	**CR**	**Cronbach's α**
Threat appraisal	TA1 TA2 TA3 TA4	0.755 0.775 0.811 0.696	0.568	0.840	0.748
Coping appraisal	CA1 CA2 CA3 CA4	0.763 0.707 0.735 0.807	0.541	0.825	0.717
Perceived benefits	PB1 PB2 PB3	0.851 0.841 0.867	0.727	0.889	0.813
Perceived risks	PR1 PR2 PR3	0.861 0.853 0.854	0.733	0.892	0.818
Trust in Physicians	TIP1 TIP2 TIP3	0.877 0.882 0.869	0.767	0.908	0.849
Trust in Applications	TIA1 TIA2 TIA3	0.930 0.924 0.919	0.854	0.946	0.915
Privacy disclosure intention	PDI1 PDI2 PDI3	0.862 0.861 0.857	0.750	0.900	0.833

AVE, Average Variance Extraction; CR, Composite Reliability.

**Table 5:** Reliability and validity analysis results.

**Construct**	**TA**	**CA**	**PB**	**PR**	**TIP**	**TIA**	**PDI**
TA	**0.752**						
CA	0.630	**0.735**					
PB	0.572	0.646	**0.853**				
PR	0.528	0.607	0.548	**0.856**			
TIP	0.406	0.529	0.426	0.400	**0.876**		
TIA	0.323	0.377	0.570	0.416	0.267	**0.924**	
PDI	0.472	0.647	0.522	0.414	0.650	0.577	**0.866**

The diagonal data in the table (bolded) are the square root of the mean-variance extracted for each variable.

### Hypothesis testing results

**Figure 2:**
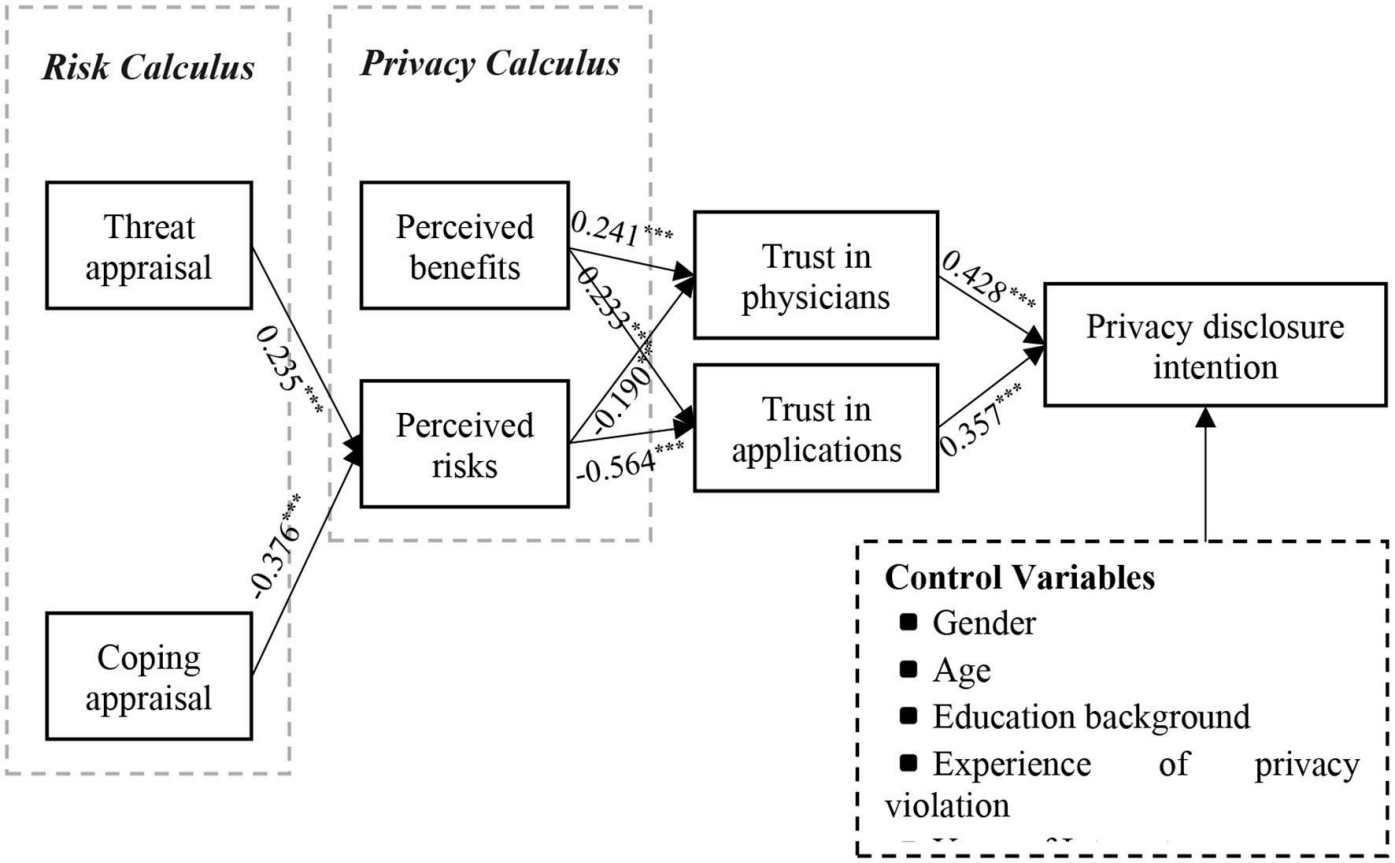
SEM analysis of research model.

In this paper, the measurement model was evaluated and analyzed using SmartPLS 4.0. Where *R*^2^ denotes the proportion of the variance of the dependent variable explained by the exogenous variables. *R*^2^ > 0.20 indicates that the endogenous variables are better explained. The results show that the *R*^2^ for trust in physicians, trust in applications, perceived risk, and privacy disclosure intention are 0.250, 0.533, 0.277 and 0.509; the adjusted *R*^2^ are 0.246, 0.531, 0.274 and 0.500, respectively, which indicate that the model has a good explanatory effect on the dependent variables. The results of model hypothesis testing are shown in Table 6 and Figure 2.

**Table 6:** Results of hypothesis testing.

**Hypotheses**	**Path coefficients**	**Mean**	**S.D**.	**T statistics**	***P–*values**
H1: TIP → PDI	0.428	0.429	0.039	10.875	0.000
H2: TIA → PDI	0.357	0.355	0.039	9.149	0.000
H3a: PB → TIP → PDI	0.103	0.104	0.031	3.277	0.001
H3b: PB → TIA → PDI	0.083	0.084	0.024	3.441	0.001
H3c: PR → TIP → PDI	−0.081	−0.082	0.029	2.774	0.006
H3d: PR → TIA → PDI	−0.201	−0.199	0.031	6.438	0.000
H4: PB → TIP	0.241	0.240	0.065	3.707	0.000
H5: PB → TIA	0.233	0.236	0.062	3.792	0.000
H6: PR → TIP	−0.190	−0.190	0.062	3.070	0.002
H7: PR → TIA	−0.564	−0.559	0.054	10.399	0.000
H8: TA → PR	0.234	0.237	0.054	4.324	0.000
H9: CA → PR	−0.376	−0.377	0.048	7.859	0.000
**Control variable**					
Gender	0.043	0.041	0.079	0.544	0.586
Age	0.021	0.001	0.035	0.435	0.696
Education background	0.005	0.005	0.036	0.142	0.887
Experience of privacy violation	−0.160	−0.159	0.072	2.208	0.027
Years of internet use	0.226	0.224	0.040	5.713	0.000

SD, Standard deviation.

During the risk calculation, the user's perceived threat appraisal in the OHCs had a significant positive effect on perceived risk (β = 0.234, *p* < 0.000), while the coping appraisal had a significant negative effect on perceived risk (β = −0.376, *p* < 0.000). Therefore, H1 and H2 are supported. During the privacy calculus, there is a positive effect of the user's perceived benefit in OHCs on the trust in both the physician (β = 0.241, *p* < 0.000) and the application (β = 0.233, *p* < 0.000). Therefore, H3 and H4 are supported. H5 and H6, examine the relationship between users' perceived risk in OHCs on trust in physicians (β = −0.190, *p* < 0.05) and applications (β = −0.564, *p* < 0.000). The results show that users' perceived risk in OHCs has a negative effect on both trust in physicians and trust in applications. That is, H5 and H6 were supported. This is consistent with Acquisti (73) and Smith et al. (74). In trust theory, user trust in physicians (β = 0.428, *p* < 0.000) and trust in applications (β = 0.357, *p* < 0.000) both have positive effects with privacy disclosure intentions. Therefore, H7 and H8 are supported. This is consistent with the findings of Zhang et al. (2). Furthermore, by comparing the effect sizes of users' trust in physicians and trust in applications, it can be found that the effect of users' trust in physicians far exceeds the effect of trust in applications. In other words, in OHCs, although users' trust in both physicians and applications has a significant effect on their willingness to disclose privacy, strong trust in physicians plays a dominant role in promoting users' willingness to disclose privacy.

### Mediating effects

In this paper, Bootstrap testing is performed using the mediated utility analysis model proposed by Preacher et al. (64) and Hayes (65). Bootstrap testing generates a series of empirical distributions of the statistics to be tested by repeatedly sampling a given dataset with put-back to create multiple simulated datasets, which allows the calculation of standard errors, the construction of confidence intervals, and the testing of multiple types of sample statistics, providing a good idea for solving small sample estimation. The Path coefficients were tested at 95% confidence intervals using SmartPLS4.0 with 5000 bootstrap samples. The means and significance levels of the path coefficients are shown in Figure 2. According to bootstrap testing, users' trust in physicians does play a mediating effect in the path of the relationship between perceived benefits and perceived risks on their willingness to disclose privacy. The confidence intervals of the Bootstrap test were [0.046, 0.169] and [−0.145, −0.028], respectively, without 0. This indicates that H3a and H3b are supported. Also, trust in the application plays a mediating effect in the path of the relationship between perceived benefits and perceived risks on their willingness to disclose privacy. The confidence intervals of the Bootstrap test are [0.038, 0.133] and [−0.261, −0.139], respectively, without 0. This indicates that H3c and H3d are supported. It can thus be shown that users' trust in physicians/applications plays a mediating effect between perceived benefits/perceived risks and privacy disclosure intentions.

## Discussion

### Theoretical contributions

First, this paper combines risk calculus and privacy calculus to construct a privacy dual-calculus model, which makes up for the deficiency of underestimating perceived risk in the previous privacy calculus process and expands the study of users' perceptions of risk when making privacy decisions. Previous studies have mostly analyzed users' motivation for privacy disclosure from the perspective of privacy calculus, arguing that their privacy disclosure decisions are made based on the difference between the perceived expected benefits and risks in the disclosure process. That is, when the perceived benefits exceed the perceived risks, users disclose privacy information; conversely, they do not, which is consistent with the findings of Dinev and Hart (12). This implies that users in OHCs need to make fully rational judgments about perceived benefits and perceived risks in order to determine the “benefit-risk” trade-off. However, individuals are bound by multiple cognitive biases in their decision-making and are unable to make fully rational privacy decisions. Because the limited rationality of users limits their ability to process the information they obtain, they rely on simplified mental models, approximation strategies, and heuristics when making decisions. In addition, Barth and De Jong (17) found that users have little access to information relevant to assessing privacy risks or irrelevant to users' privacy protection due to their own limited cognition or information asymmetry, which can lead to their inability to perform a complete risk-benefit calculation and ignore or underestimate the risks by perceiving the benefits more obviously (66). According to PMT, users' perception of risk is determined by both their assessment of external threats and their own ability, and using privacy computation theory alone does not accurately reflect users' perception of risk when making privacy decisions. Therefore, this paper combines risk calculus and privacy calculus to construct a privacy dual-calculus model, which on the one hand compensates for the shortcomings of privacy calculus in ignoring or underestimating perceived risks, and on the other hand, provides a new perspective for understanding the process of the role of privacy disclosure intentions of users in OHCs.

Second, this paper explores the effects of two types of trust on users' privacy disclosure intentions. Although user trust in both doctors and applications significantly affects their privacy disclosure intentions, strong trust in doctors plays a more important role in promoting users' privacy disclosure intentions. In general, trust indicates people's willingness to rely on or to be vulnerable to other parties (29, 30), and it is often seen as an important factor in building and strengthening relationships and facilitating user information exchange. However, existing research has mainly focused on the effects of trust in the applications/services, as well as its predictors (4, 31, 32), while few studies have mostly conceptualized user trust in OHCs. For example, trust between patients and doctors, trust between patients and technology, or trust between patients and applications. Accordingly, this paper refines the different trust transfer processes of users in OHCs by dividing users' trust in OHCs into trust in physicians and trust in applications. The comparison reveals that the influence effect of user trust in physicians far exceeds that of trust in applications. In other words, although users' willingness to disclose privacy in OHCs is affected by both trust in physicians and trust in applications, the effect of trust in physicians is stronger in promoting users' willingness to disclose privacy. This is because in OHCs, both trust in physicians and trust in applications play an important role in users' final decisions. However, with the continuous improvement of privacy protection laws and platform governance norms, and the development of online health community applications toward scale, standardization and standardization, the difference in trust brought by different online health community applications to users is gradually shrinking. At the same time, the goal of users is to solve health-related problems, and the personal attributes and professional labels of physicians are likely to be the basis for their information disclosure decisions, while the trust back in the applications will also strengthen this willingness of users.

Finally, this paper verifies the mediating effect of user trust in physicians and applications between privacy calculus and privacy disclosure intention. In OHCs, users' decision to disclose privacy information comes from a balance between perceived benefits (e.g., material benefits, social benefits, and service experience, etc.) and perceived risks (e.g., privacy leakage, privacy violation, inappropriate access, and secondary use, etc.). When users can conveniently use various convenient services provided by online health applications, on the other hand, they can learn the personal information of doctors related to their consultation needs and have one-on-one communication with them. This can largely increase users' trust in doctors and applications and help increase their willingness to disclose their privacy. When users perceive physical, psychological, privacy, functional, social, and financial risks on healthcare websites, they usually choose to stop trusting the websites and the doctors on the websites to avoid losses for the sake of protecting their own interests, which is an instinct of users.

### Practical implications

First, users of OHCs need to be aware that relying on privacy calculations alone is not sufficient for making privacy disclosure decisions about health information. This is because privacy calculations often underestimate the risks of disclosure and overestimate the benefits to be gained. Therefore, privacy calculations should be combined with risk calculations to assess the net risk of making online decisions. Unlike traditional e-commerce communities, OHCs encourage patients to express themselves freely and share information. Instead of one-to-one communication between patients and doctors, there is a one-to-many exchange with other patients and physicians through OHCs. Therefore, its information flow is interactive and multilateral. This provides convenience to patients while also increasing the frequency of information disclosure and widening the channels of information disclosure, increasing the concern about the privacy of health information disclosure. Therefore, for patients, it is currently less expensive for users to learn about OHCs and privacy-related information through the Internet, and the amount of access to information and cognitive benefits are greater, which plays an important role in improving their own medical knowledge and privacy awareness. By improving their awareness of OHCs and their ability to discriminate, users can effectively and rationally evaluate risk information from outside sources and decide whether to take protective or unprotective actions.

Second, OHCs' platform operators need to understand that patients will evaluate the potential benefits and potential risks of disclosing their health privacy information, which plays a critical role in developing user trust in the physician and the application. It is important for OHCs to offer perceived rewards to users who disclose personal privacy information, which is in line with Acquisti and Smith et al. (67, 68). Therefore, for OHCs, platform operators can attract users' engagement by providing an easy-to-use experience, good online word-of-mouth, and high-quality medical services, thus forming an initial trust in the website. Next, the user's perception of risk usually comes from a lack of knowledge or information asymmetry. At this point, platform operators should, on the one hand, establish sound interactive communication channels between users and users and between users and doctors; on the other hand, they should improve the transparency of policies on application collection, use and protection of users' privacy information and the level of disclosure of doctors' personal information and expertise. These measures can allow patients to form a subjective anchor mentality of trust in the reliability of OHCs and the authenticity and expertise of physicians. Furthermore, platform operators should place a high priority on patients' perception of the application and doctors' service experience, actively focus on users' individual needs and precise services, and introduce high-quality medical information and high-level and highly qualified doctors into the platform for medical services. It is important to provide targeted services to patients and help them solve their problems in seeking medical treatment so that users can form long-term and stable trust in applications and physicians from the online health community applications and physicians.

Finally, platform operators must clearly recognize that users' trust in online health community applications and physicians is a prerequisite to motivate them to disclose health privacy information. And, to a greater extent than users' trust in the application, their trust in the physician will enhance their willingness to disclose privacy. In many cases, users are unfamiliar with and have no experience interacting with OHCs and physicians. As a result, they often need to reduce the high perceived risk and uncertainty of interacting with unfamiliar targets based on valuable and reliable services offered by the app as well as cues (e.g., others' experiences, physician information, platform reputation, etc.). Building one's perceived trust based on application cues is the process by which trust is transferred between subjects. In this study, operators of online general wellness communities need to recognize that when patients seek help, their choice of applications and doctors requires the transfer of trust mechanisms, which depend on the balance between perceived benefits and risks to users. Users' expectations and confidence in outcomes can be met by enhancing privacy rules, granting benefits, offering personalized services, and free trials to enhance their perceived benefits while reducing their perception of risk. In addition, platform operators should pay attention to the introduction of high-level physicians and the construction of high-level medical teams to win the trust of users with high-quality medical service levels and professional teams of physicians.

## Conclusions

This study combines risk calculus and privacy calculus theories to construct a dual-calculus model, which divides trust into trust in physicians and trust in applications, in order to explore the intrinsic motivation and decision-making mechanism of users' participation in privacy disclosure in OHCs. There are three main contributions: first, this paper theoretically bridges the gap that privacy computing often underestimates perceived risks, which is helpful to help patients in OHCs make more reasonable privacy computing assessments; second, this paper conceptualizes users' trust in OHCs in multiple dimensions, such as trust between patients and doctors, trust between patients and technologies, or trust between patients and applications. This enriches the research on trust in OHCs; third, this paper reveals the influencing factors and decision transmission mechanisms of user privacy information disclosure in OHCs, which provides guidance for platform developers of OHCs to develop reasonable privacy policies and health information protection mechanisms.

The findings show, first, that in the risk calculus, users' perceived threat appraisals in OHCs have a significant positive effect on perceived risk, while coping appraisals have a significant negative effect on perceived risk. Moreover, the effect of users' trust in physicians far exceeds the effect of trust in applications. Second, in the privacy calculus, users' perceived benefits in OHCs had a positive effect on both physicians and trust in applications. In contrast, the user's perceived risk has a negative effect on both trust in the physician and applications. This suggests that users' trust in physicians and applications is a prerequisite for their willingness to disclose health information. Therefore, the platform developers of OHCs should effectively fulfill the responsibility of health care organizations to provide quality medical services while protecting patients' health information from being violated. Finally, users' trust in physicians/applications plays a mediating effect between perceived benefits/perceived risks and privacy disclosure intentions.

There are also some limitations in this study. First, it is difficult to obtain data on the disclosure of user health information confined to OHCs. This paper relies on self-reported questionnaires to discuss users' privacy disclosure intentions without analyzing their actual privacy decision-making behaviors based on real data, which may be influenced by the privacy paradox to some extent (55–57). Second, users' privacy disclosure intentions in OHCs are subject to a variety of boundary conditions, such as the level of privacy of the disease, the physician-patient relationship, the severity of the disease, the difference in physicians' titles, and the consultation prices set by the physicians. However, this paper does not address this due to data availability issues. Finally, user privacy disclosure is a complex topic, and its behavioral decisions can be divided into three types: “risk-benefit” calculations based on rational assumptions, biased estimates of privacy decision risks influenced by limited rationality, and perceived benefit-based assessments with no privacy decision risks. In this paper, only privacy and risk calculations are considered, and the latter two privacy decision models are not explored (17). Third, our data originated from only one country (China), which could potentially affect the global generalizability of the study's findings. This is because there are significant differences in attitudes toward privacy in different countries (58). Future research could collect data on users' real behaviors in OHCs or use randomized scenario experiments, which could eliminate other factors to make the results more generalizable. At the same time, more moderating variables can be introduced to reveal the boundary conditions of users' privacy disclosure behaviors in OHCs. In addition, cognitive heuristics, affective information theory, optimistic bias theory, third-person effect, self-control bias theory, immediate gratification, hyperbolic discounting theory, prospect theory, and learned helplessness can be combined to provide more diverse explanations for the motivations and underlying mechanisms of users' privacy disclosure intentions/behaviors in OHCs. Meanwhile, the intrinsic motivation and psychological mechanisms of users in OHCs for health information disclosure in different national contexts can be studied from a cross-cultural perspective in the future.

## Data availability statement

The raw data supporting the conclusions of this article will be made available by the authors, without undue reservation.

## Author contributions

Conceptualization and writing—original draft preparation: ZS. Methodology and funding acquisition: GZ. Conceptualization and investigation: ZW. Data collection: SG and WL. Writing—original draft preparation and formal analysis: KW. All authors contributed to the article and approved the submitted version.
